# Proximity to a hazardous waste thermal treatment facility alters human physiology: a community-driven pilot study

**DOI:** 10.3389/ebm.2025.10655

**Published:** 2025-08-15

**Authors:** Avinash Kumar, Chuqi Guo, Qudus Sarumi, Christopher Courtney, Shawn Campagna, Jennifer Richmond-Bryant, Stephania A. Cormier

**Affiliations:** ^1^Department of Biological Sciences, Louisiana State University, Baton Rouge, LA, United States; ^2^ Pennington Biomedical Research Center, Baton Rouge, LA, United States; ^3^Department of Forestry and Environmental Resources, North Carolina State University, Raleigh, NC, United States; ^4^Department of Chemistry, University of Tennessee, Knoxville, TN, United States; ^5^Biological and Small Molecule Mass Spectrometry Core, University of Tennessee, Knoxville, TN, United States

**Keywords:** metabolomics, open burn, hazard waste remediation, environmental exposure, oxidative stress

## Abstract

Open burn/open detonation (OB/OD) disposes of explosive waste via uncontrolled combustion, releasing harmful pollutants like toxic gases and particulate matter. Colfax, Louisiana, houses the nation’s only commercially OB/OD thermal treatment (TT) facility, raising concerns about environmental and public health impacts due to its emissions. In this exploratory pilot study, we investigated metabolic alterations indicative of potential health impacts from exposure to emissions from a TT facility through an untargeted metabolomics analysis of urine samples obtained from local residents. Urine samples were collected from 51 residents living within a 30-km radius of the facility, with proximity, race, and sex as key variables. Samples were analyzed using ultra-high-performance liquid chromatography coupled with high-resolution mass spectrometry (UHPLC-HRMS) to identify metabolic alterations and potential biomarkers of exposure. A total of 217 metabolites were identified, with significant differences in abundance based on proximity to the facility. Key metabolic pathways affected included energy metabolism, amino acid metabolism, and oxidative stress-related pathways. Metabolites associated with oxidative stress, such as glutathione sulfonamide (GSA), were elevated in individuals residing closer to the facility, indicating increased oxidative stress. Alterations in the glutathione/glutathione disulfide (GSH/GSSG) ratio further highlighted redox imbalances. Pathway enrichment analyses revealed perturbations in glycolysis, citric acid cycle, sulfur metabolism, and nucleotide metabolism, which are linked to critical biological functions like energy production and DNA repair. Notable differences in metabolite profiles were also observed between sexes and racial groups, pointing to the interplay of intrinsic biological and environmental factors. These findings demonstrate that exposure to emissions from the TT facility may have significant impacts on human health, including disruptions in cellular metabolism and increased oxidative stress. Further research is crucial to understand the long-term health implications of these metabolic alterations and to develop strategies to mitigate the environmental and health risks associated with this facility.

## Graphical Abstract

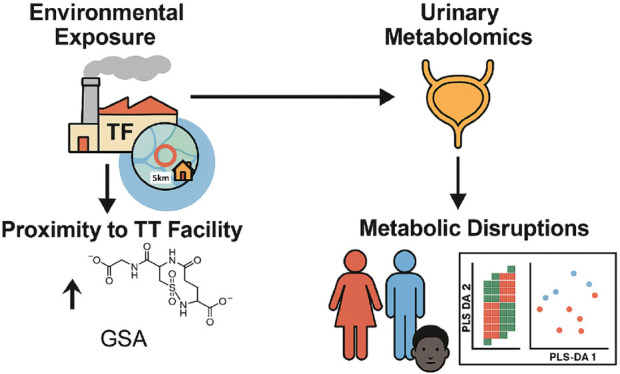

## Impact statement

This study provides critical insights into how environmental exposure to emissions from thermal treatment (TT) facilities can disrupt human metabolism at a cellular level, using urinary metabolomics as a non-invasive monitoring approach. By linking metabolite alterations, particularly in energy, amino acid, and nucleotide pathways, with proximity to the TT facility, this work highlights a potential biochemical signature of exposure. Notably, elevated levels of glutathione sulfonamide (GSA) and disrupted antioxidant balance underscore oxidative stress as a key biological response. The integration of demographic factors such as sex and race adds an important dimension to understanding individual susceptibility. This research advances the field by establishing metabolomics as a sensitive biomonitoring approach for environmental health assessment and by identifying novel metabolite markers of exposure. These findings offer a foundation for future public health strategies, regulatory frameworks, and longitudinal studies, ultimately enhancing our understanding of environmental toxicology and personalized exposure risk evaluation.

## Introduction

Colfax, a small rural town in central Louisiana, United States, has a population of 1,442 as of 2023, with 61.1% identifying as Black or African American and 33.1% as White [1] compared with the Parish as a whole, with a population of 22,123 (14.8% identify as Black or African American, 78.2% as White [2]). The community faces economic challenges, with a median individual income of $20,192 and about 35% of residents living below the poverty line. Colfax is notable for housing the country’s only commercially operated open burn/open detonation (OB/OD) thermal treatment (TT) facility. In the latest permit application, the Louisiana Department of Environmental Quality (LDEQ) ordered the TT facility to cease OB/OD operations in December 2023 until a closed-burn facility could be constructed; however, the facility is currently appealing this decision in court and remains in operation. The TT facility, in operation since 1985, was previously permitted to treat a wide variety of waste streams, including military ordnances, cylinders, explosives, and propellants, as well as hazardous materials from Superfund sites, fireworks, and ammonium perchlorate [3]. These materials contain toxic components such as metals, explosives, and organohalogens. Without containment or emission control measures, OB/OD operations at the facility released particulate matter (PM) directly into the atmosphere, raising concerns about toxic exposure risks for the Colfax community. Community members living in close proximity to the TT have reported a range of health issues, including thyroid disease, respiratory and cardiovascular disease, skin damage, and cancer [4]. Exposure to certain toxic materials or emitted pollutants has been shown in studies to cause similar health effects [5–16], further amplifying concerns about the impact of the facility’s operations on community health.

Metabolomic analysis [17, 18] offers a method for understanding connections between Colfax community members’ exposures to PM generated by OB/OD and health outcomes [4, 19] The metabolome is a highly dynamic and individualized biochemical fingerprint that responds rapidly to internal and external influences. It provides a comprehensive snapshot of physiological states of individuals at a particular time, making it a diagnostic tool for changes observed in response to environmental exposure [20]. Metabolites present in tissues or body fluids can serve as intermediate or final products of cellular metabolism, as well as byproducts of energy-producing nutrients, intermediates in the synthesis of biological macromolecules, and waste products that influence normal cellular functions [21]. Since several biomarkers are produced through cellular metabolism and various protein activities, they can indicate the effects of functional changes triggered by external exposures, such as those from OB/OD operation, delineating the potential pathways through which hazardous exposures affect health [22, 23]. Urine is also emerging as a preferred biofluid for the noninvasive monitoring of human health because changes in secreted biomarkers may be easier to detect as there is little to no evolutionary pressure to maintain homeostasis within this waste product [24]. In fact, a recent study conducted in Shanghai, China, demonstrated that short-term exposure to PM_2.5_ can cause significant alterations in urinary metabolomic profiles, potentially leading to disruptions in energy metabolism, oxidative stress, and inflammation [25]. Another study evaluated metabolic response to short-term exposure to airborne PM_2.5_ and bioaerosols using gas chromatography/liquid chromatography-mass spectrometry (GC/LC–MS) with urinary samples [26]. Key findings revealed that 33 out of 155 differential metabolites were associated with PM_2.5_ and bioaerosol exposure. It also revealed potential biomarkers like 16-dehydroprogesterone and 4-hydroxyphenylethanol identified for predicting PM_2.5_ - or bioaerosol-related diseases, highlighting dynamic changes in urinary metabolic profiles in response to air pollution.

Our previous field studies conducted in the same community have included reviews of corporate reports indicating that weapons having metal casings and ammonium perchlorate accelerant and other explosive materials are often burned by ignition with diesel [4], producing PM with complex composition. Measurements have detected environmentally persistent free radicals (EPFRs), metals, and polychlorinated dibenzo-p-dioxins and dibenzofurans (PCDD/Fs) in ambient PM_2.5_ and soil samples [27], with the radical electron localized on carbon with an adjacent oxygen. Residents living near the TT facility have reported several health issues, including thyroid disease, respiratory and cardiovascular conditions, skin damage, and cancer [4]. This study applied an untargeted metabolomics investigation using urine from community members residing near and potentially exposed to emissions from the TT facility in order to identify potential biomarkers of exposure and reflecting a systemic health effect that may help substantiate community members’ stated concerns about health effects. Since no established biomarkers exist for exposure to EPFRs and real-world exposures typically involve complex mixtures rather than single agents, this work represents the first attempt to identify biomarkers specific to EPFR exposure.

## Materials and methods

### Subject recruitment and sample collection

The study population consisted of residents living within a 30-km radius of the facility. Initially, 53 community members (21 male and 32 female) were recruited. However, two participants were unable to provide urine samples, resulting in a final validated sample size of 51 (21 male and 30 female) (Table 1). Participant ages ranged from 24 to 89 years, with a mean age of 65.18 +/−15.26 (SD). Among the 51 individuals providing urine samples, 20 lived within 5 km of the TT facility, and 31 lived further away. A 5 km radius was selected based on our previous work in the community, which identified this distance as encompassing the greatest concentration of reported thyroid, respiratory, and skin conditions members [4]. This boundary is also supported by empirical evidence from prior epidemiological studies demonstrating elevated health risks within similar proximities to environmental pollution sources [28, 29].

**TABLE 1:** Participant demographics.

Race	F	M	Total
Black	17	8	25
White	13	13	26
Total	30	21	51

This study was reviewed and received approval from the North Carolina State University Institutional Review Board (Protocol #25124) with a reliance agreement to Louisiana State University. All participants signed a consent form agreeing to urine collection. The consent form stipulated that the participants’ names were kept on a crosswalk document that is separate from the samples, which were assigned random ID numbers. Address, gender, and race were also recorded on the crosswalk table, and home address was used to obtain distance from the TT facility’s burn pads. To ensure confidentiality, the table was de-identified before being sent to the laboratory.

Participants were provided with a urine sample collection kit, including a sterile collection bottle, alcohol wipes, and detailed instructions that followed standard hospital practices. Study participants were furnished with instructions requesting that the urine be collected mid-stream; no special instructions for time of collection were provided. Following collection, samples were kept on ice during the collection period and were stored at −80°C immediately upon transfer to the laboratory. A chain-of-custody form was created for the urine sample collection, and a label containing the sample ID was applied to the sample and entered on the crosswalk table.

### Sample analyses

#### Metabolomics method

##### Sample preparation and extraction

Stored urine samples were thawed in a cold room at 4°C for 1 h before 100 µL aliquots were prepared for metabolite extraction following established protocols [30, 31]. Briefly, 1.5 mL of extraction solvent (20:40:40 water/methanol/acetonitrile with 0.1 M formic acid) was added to the urine samples in 2-mL microcentrifuge tubes. The tubes were vortexed thoroughly to ensure proper mixing with the extraction solvent. After mixing, the samples were chilled at −20°C for 20 min to enhance metabolite release. The samples were then centrifuged at 15,000 rpm for 5 min at 4°C to separate the supernatant, which contained the extracted metabolites, from the residue. The supernatants were carefully transferred into new microcentrifuge tubes and dried under a stream of nitrogen gas to completely remove the solvent. Once dried, the samples were reconstituted in 300 µL of LC-MS grade water, vortexed, and centrifuged again at 15,000 rpm for 5 min at 4°C to ensure homogeneity. The final extracts were aliquoted into autosampler vials for analysis using ultra-high-performance liquid chromatography coupled with high-resolution mass spectrometry (UHPLC-HRMS). Metabolites were extracted without the addition of internal standards, as the study design focused on relative quantification rather than absolute concentration measurements. Metabolite identification was achieved by matching retention times and mass-to-charge ratios (m/z) to an in-house standard library. Quantification was based on the relative abundances of detected features across sample groups. Given the absence of spiked internal standards, the data is considered semi-quantitative, suitable for comparative analyses. To evaluate extraction efficiency, blank extractions were processed in parallel using identical protocols. Non-specific signals were assessed by comparing metabolite profiles from the blank extractions with those from biological samples, allowing for blank subtraction and recovery estimation to identify background artifacts and assess extraction performance.

##### UHPLC-HRMS analysis

Metabolite separation and mass analysis were conducted at the University of Tennessee Biological and Small Molecule Mass Spectrometry Core (RRID: SCR_021368) using an UltiMate 3000 RS chromatograph (Dionex, Sunnyvale, CA, United States) coupled to an Exactive™ Plus Orbitrap mass spectrometer (Thermo Fisher Scientific, Waltham, MA, United States). A reversed-phase ion-pairing chromatographic method was employed for separation, utilizing a Synergi Hydro RP column (2.5 μm, 100 × 2.0 mm; Phenomenex, Torrance, CA, United States). The mobile phase consisted of LC-MS grade solvents composed of 97% water, 3% methanol, 11 mM tributylamine as the ion-pairing reagent, and 15 mM acetic acid, with gradient elution performed as previously described by Bazurto et al. [32]. The separation was carried out at a flow rate of 0.2 mL/min for 25 min, with the column temperature maintained at 25°C. The eluted metabolites were ionized using electrospray ionization in negative polarity mode. Mass analysis was performed using the Orbitrap mass analyzer at a resolution of 140,000, with a scan range of 72–1,000 m/z, an injection time of 100 milliseconds, and an automatic gain control (AGC) target of 3 × 10^6^, as detailed in the protocol by Bazurto et al. [32].

##### Data analysis

The Xcalibur (RAW) files generated from the UHPLC-HRMS analysis were converted to mzML format using the msconvert software, a tool from the ProteoWizard package. This conversion transformed the profile data into centroided data, improving their suitability for downstream analysis. The centroided data were then uploaded to the Metabolomic Analysis and Visualization Engine (MAVEN) [33, 34], an open-source software tool developed at Princeton University. MAVEN was utilized for visualization of extracted ion chromatograms (EICs), peak feature selection, peak area integration, and metabolite identification using an in-house standards library. MAVEN automatically corrected for retention time nonlinearity and aligned peak areas across all samples. Metabolites were identified by comparing chromatographic retention times ±2 min, peak shapes, signal-to-noise ratios, and exact masses within a ±5 ppm mass tolerance to an in-house standard library. Identification was further validated by comparing the natural isotopic abundance patterns of the compounds. Peak intensity data tables were generated from MAVEN for statistical analysis. Metabolite intensities were normalized by the sum to account for differences in the initial concentrations of urine samples and by volume to express metabolite ion counts per mL of urine. Due to the known biological variability of creatinine levels—which can be influenced by factors such as age, sex, muscle mass, diet, and hydration status—samples were normalized using the sum (or Total Useful Signal) approach. This data-driven method assumes that most metabolite intensities remain relatively constant across samples and has been shown in multiple studies to outperform creatinine normalization in untargeted metabolomic analyses [35, 36]. A heatmap was generated using a custom script in R Studio (version 4.2.1, RStudio Team, 2022, Boston, MA, United States). Further downstream data analysis was performed using Metaboanalyst 6.0. Variance filtering was applied using the interquartile range (IQR), and the data were log-transformed for better visualization and Pareto-scaled. Partial Least Squares-Discriminant Analysis (PLS-DA) was conducted to visualize metabolic profile differences across sample groups. Metabolites showing significant differences and a Variable Importance in Projection (VIP) score greater than 1 were selected for Kyoto Encyclopedia of Genes and Genomes (KEGG) pathway enrichment analyses. All statistical analyses were considered significant at P < 0.05, and pathway significance was determined at a False Discovery Rate (FDR) cutoff of <0.05.

## Results

Metabolomic analysis was conducted to assess if proximity to the TT facility and exposure to emissions altered the metabolome, as the metabolome is directly related to phenotype. A total of 217 metabolites were identified from 51 urine samples. Among these, 10 metabolites belonged to the vitamin superclass, 2% were neurotransmitters, 9% were related to amino acid metabolism, and 8% were involved in glycolysis, gluconeogenesis, and pentose phosphate pathways. Additionally, 22% were nucleosides, nucleotides, and analogs, 6% were lipid metabolites, 6% were carbohydrates and conjugates, 3% were citric acid cycle metabolites, and 6 metabolites were categorized under bile acids and bile salts. Most of the identified compounds were amino acid precursors and derivatives, which accounted for approximately 25% of the total metabolites, along with carbohydrates involved in nutrient metabolism.

**FIGURE 1:**
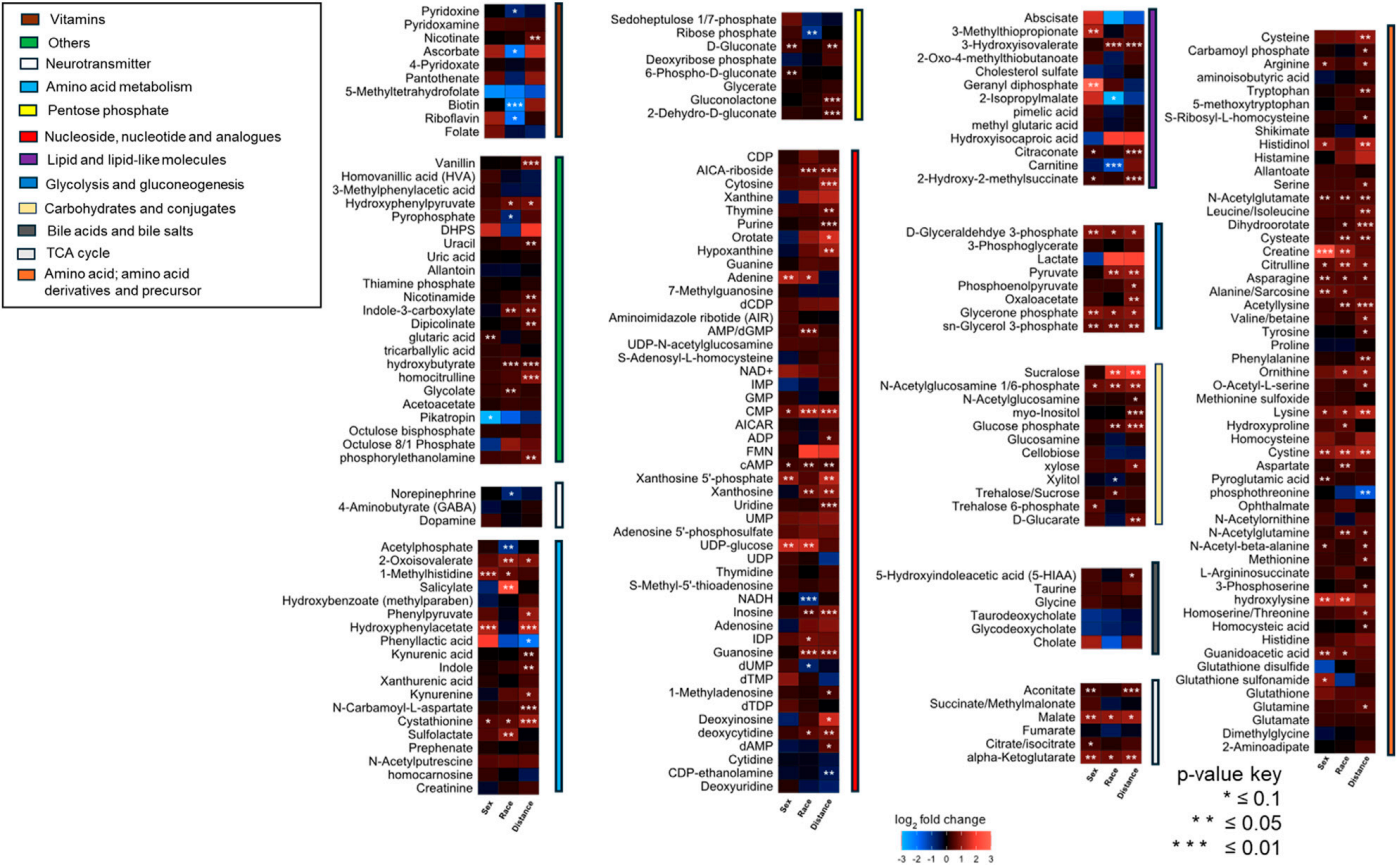
A heatmap illustrating fold changes in metabolite abundance, organized by compound class, across sample groups stratified by sex, race, and proximity to the facility. The fold changes include Female/Male, Black/White, and Less than 5 km/Greater than 5 km (n = 51). Statistically significant differences are indicated by asterisks (*P < 0.1, **P < 0.05, ***P < 0.01). Metabolite abundance is represented using a color gradient, with red indicating a higher abundance and blue indicating lower abundance in female for sex-based comparison, Black individuals for race-based comparison, or individuals residing less than 5 km from the facility for proximity-based comparison.

Significant differences in metabolite abundance were observed across urine samples collected from community members (positive fold change ≥2, negative fold change ≤0.5 and P-value cut-offs <0.1). To determine if proximity to the TT facility resulted in metabolome alterations, fold changes were calculated for metabolites between individuals residing less than 5 km vs. those greater than 5 km from the TT facility’s burn pads, sex (male vs. female), and race (Black vs. White). Based on our previous discussions with residents [4], a cut-off of 5 km was chosen to differentiate residents living in the Rock community or those in similar proximity to the east, west, and north, from those in the surrounding area living further away. The relative abundance of metabolites varied significantly across these categories, with the greatest differences observed between the less than 5 km and greater than 5 km groups. Most metabolites were found to be higher in abundance in individuals residing closer to the facility (less than 5 km) compared to those farther away (Figure 1).

A p-value threshold of <0.1 was adopted to allow for broader detection of metabolite-level differences in this exploratory study, where the primary aim was to identify potential trends and biologically relevant patterns across sample groups. This approach has been used in similar untargeted metabolomics studies where a balance between sensitivity and specificity is critical in the initial discovery phase [37]. To address the increased risk of false positives associated with this more lenient threshold, false discovery rate (FDR) correction was applied to pathway enrichment analyses, where the biological interpretation of grouped metabolites is most meaningful and informative. The combination of a relaxed p-value threshold and FDR correction at the pathway level provides a balanced strategy for exploratory data interpretation while minimizing the likelihood of spurious associations. In future studies with larger sample sizes, we intend to apply stricter significance thresholds and uniform correction strategies.

**FIGURE 2:**
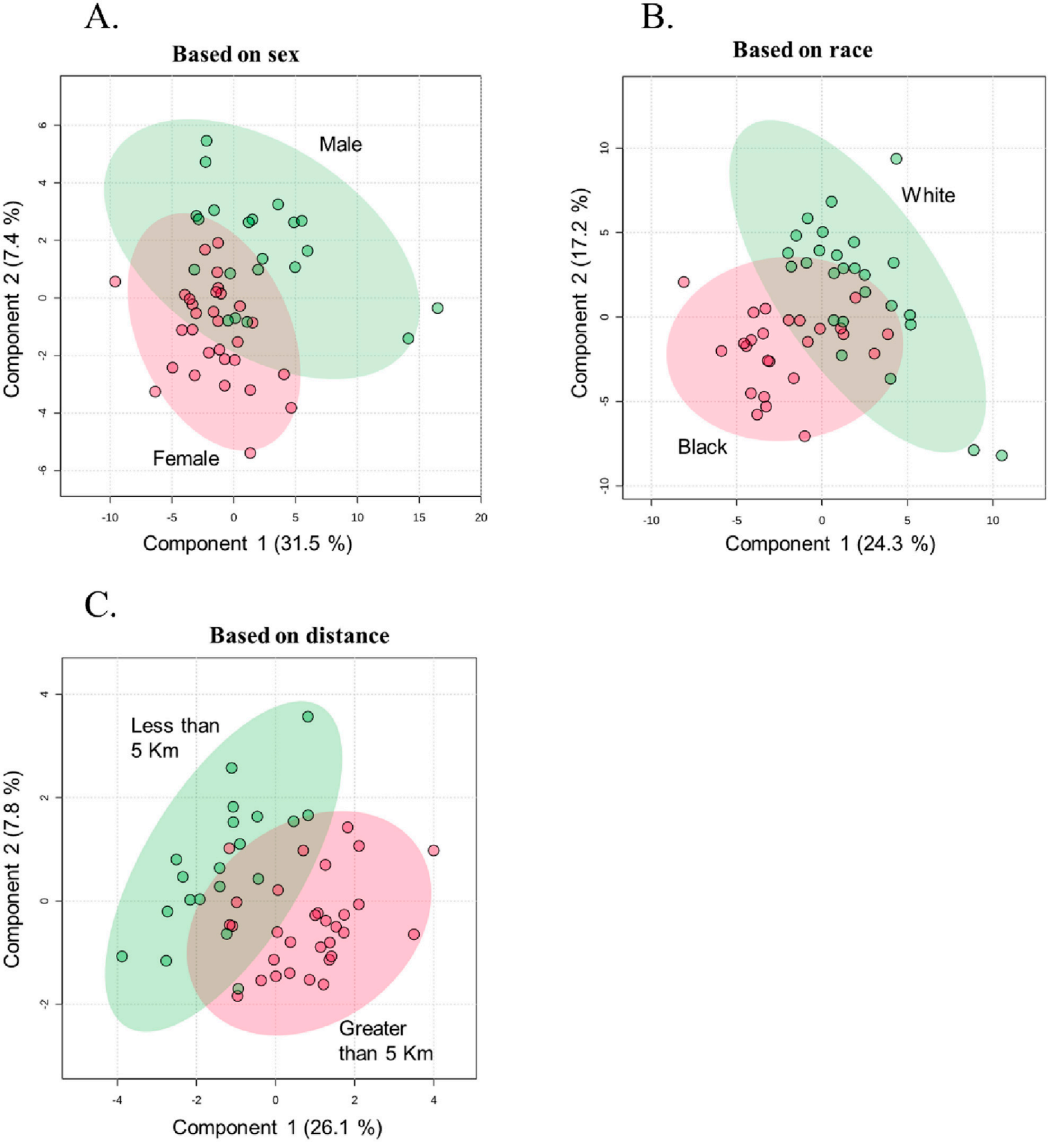
Partial Least Squares-Discriminant Analysis (PLS-DA) plots illustrating metabolic profile separations with 95% confidence intervals, grouped by: **(A)** Sex (Female vs. Male), **(B)** Race (Black vs. White), and **(C)** Distance from the facility (Less than 5 km vs. Greater than 5 km) (n = 51).

**FIGURE 3:**
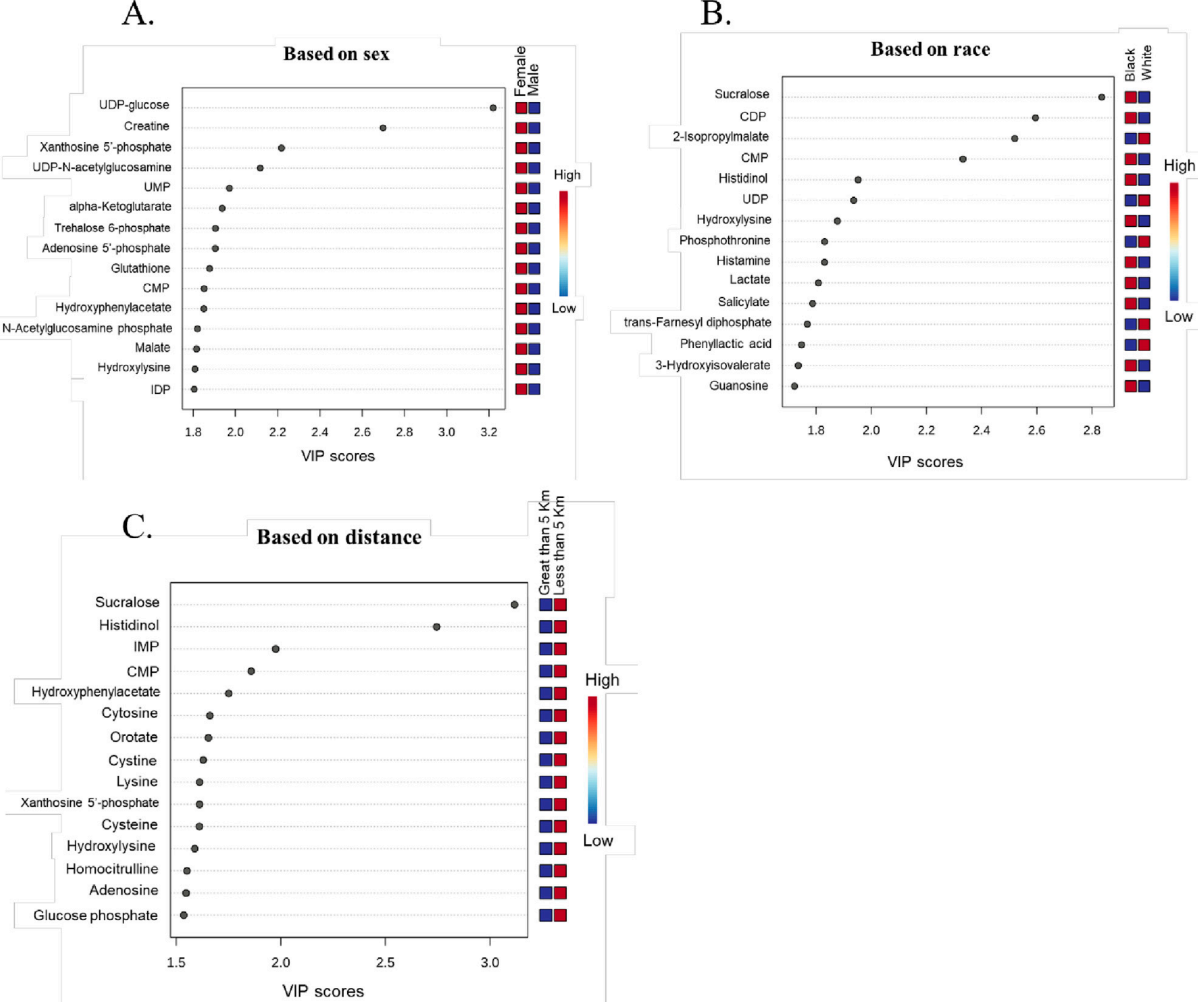
Variable Importance in Projection (VIP) score plots highlighting metabolites with 15 highest VIP scores. All metabolites with a VIP score greater than 1 significantly contribute to the observed group separations. These metabolites drive the differences across **(A)** Sex (Female vs. Male), **(B)** Race (Black vs. White), and **(C)** Distance from the facility (Less than 5 km vs. Greater than 5 km) (n = 51).

**FIGURE 4:**
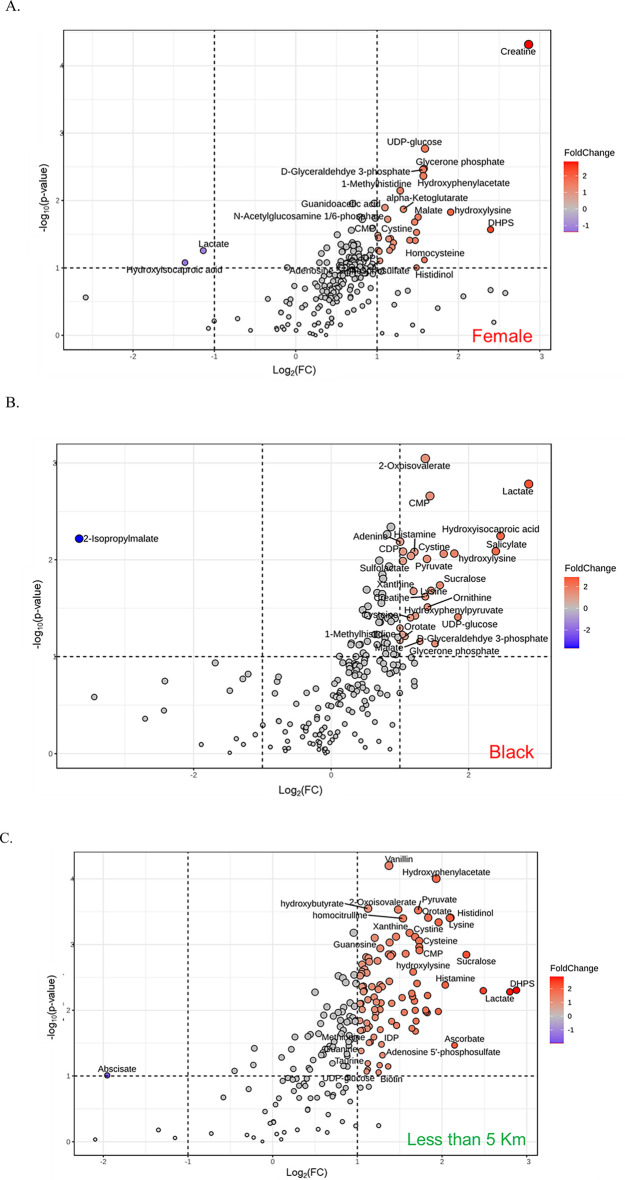
Volcano plots illustrating the differentially abundant metabolites across groups: **(A)** Sex (Female vs. Male), **(B)** Race (Black vs. White), and **(C)** Distance from the facility (<5 km vs. >5 km). Metabolites with a fold change ≥2 and P-value <0.1 are highlighted with red spot, while those with a fold change ≤0.5 and P-value <0.1 are highlighted with blue spot.

Partial least squares discriminant analysis (PLS-DA), a supervised multivariate chemometric tool, was employed to cluster sample groups based on similarities in their metabolic profiles. A 2D PLS-DA score plot was generated to illustrate differences in metabolic profiles between groups by sex (Figure 2A), race (Figure 2B), and proximity to the facility (Figure 2C). The evident separation in the plots indicates distinct differences in metabolic profiles among the groups, suggesting variations in metabolite abundance and distinct metabolic activities, as reflected by clustering along the components. Additionally, the variable importance in projection (VIP) scores are assigned to each metabolite to indicate the extent to which each metabolite contributes to the observed separation in the PLS-DA model. Metabolites with a VIP score greater than 1 are key drivers of global metabolome profile differences between groups. VIP score plots for sex (Figure 3A), race (Figure 3B), and proximity to the facility (Figure 3C) illustrate the metabolites that significantly influence the separation in the PLS-DA plots, underscoring their pivotal role in delineating group differences. To further explore metabolic differences, volcano plots were used to visualize fold changes and statistical significance across the groups. The volcano plot for the female vs. male comparison (Figure 4A) highlights metabolites with fold changes greater than or equal to 2 in red, alongside metabolites with statistical significance (p ≤ 0.1). Similarly, the volcano plot for the race comparison (Figure 4B) shows metabolites with statistically significant upregulation or downregulation. Proximity to the facility revealed the most pronounced differences, with the volcano plot (Figure 4C) showing several metabolites with fold changes that were substantially elevated in the <5 km group compared to the >5 km group.

**FIGURE 5:**
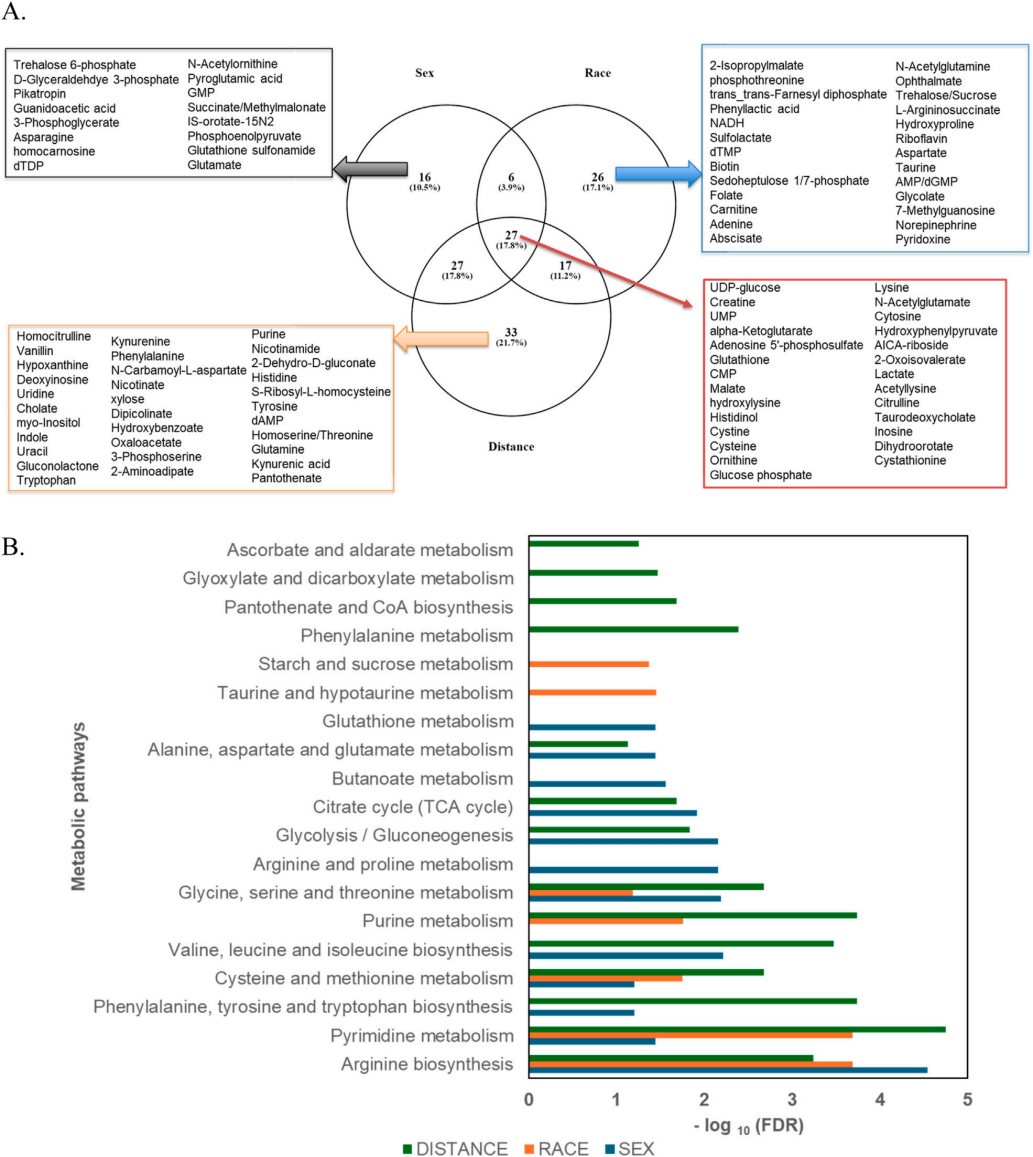
**(A)** Venn diagram depicting the distribution of differentially abundant metabolites uniquely identified in each group—Sex (n = 16), Race (n = 26), and Distance (n = 33)—as well as the metabolites shared across all groups (n = 27). **(B)** Clustered bar graph illustrating the enriched metabolic pathways across the sample groups, with logarithmically transformed FDR values. Pathway significance was determined using P-values, with a false discovery rate (FDR) threshold of <0.05.

A Venn diagram (Figure 5A) was used to analyze the unique and shared metabolites among the sample groups categorized by sex, race, and proximity to the thermal treatment facility. A total of 27 metabolites were common across all three groups. The number of unique metabolites varied, with the proximity-based comparison exhibiting the highest number (33 unique metabolites), followed by the race-based comparison (26 unique metabolites), and the sex-based comparison (16 unique metabolites). 10.5% of the metabolites were unique to the sex-based comparison, and they include glutathione sulfonamide, phosphoenolpyruvate, and glutamate. The race-based comparison revealed 17.1% unique metabolites, such as 2-isopropylmalate, phenyl lactic acid, NADH, N-acetyl glutamine, trehalose/sucrose, aspartate, taurine, and glycolate. The proximity-based comparison, which focused on individuals residing less than 5 km versus greater than 5 km from the facility, identified 21.7% unique metabolites. These included uridine, cholate, myo-inositol, indole, uracil, gluconolactone, phenylalanine, N-carbamoyl-L-aspartate, oxaloacetate, purine, nicotinamide, nicotinamide, S-ribosyl-L-homocysteine, tyrosine, homoserine/threonine, and glutamine (Table 2). Additionally, 3.9% of metabolites were shared between the sex and race groups, 17.8% between the sex and proximity groups, and 11.2% between the race and proximity groups. A total of 17.8% of metabolites were shared across all three groups. These included UDP-glucose, creatine, UMP, alpha-ketoglutarate, glutathione, CMP, malate, cystine, cysteine, glucose phosphate, lysine, cytosine, lactate, taurodeoxycholate, and cystathionine. Enrichment analysis for metabolic pathways across the sample groups was visualized in a clustered bar graph (Figure 5B), with the pathways’ significance evaluated based on their false discovery rate (FDR) plotted on a logarithmic scale. The bar graph revealed that the most significant pathways in the proximity-based groups were pyrimidine and purine metabolism, as well as phenylalanine, tyrosine, and tryptophan biosynthesis. In the race-based comparison, arginine biosynthesis and pyrimidine metabolism were prominent, while glycolysis and arginine biosynthesis pathways showed significant enrichment in the sex-based groups. These findings highlight the distinct metabolic activities and pathway perturbations across the groups, potentially linked to demographic and environmental factors.

**TABLE 2:** Differential abundance of metabolites across sample groups.

List of metabolites that are uniquely present in sample groups categorized by sex, race, and proximity to the thermal treatment facility			
Sex-based unique metabolites	Race-based unique metabolites	Distance-based unique metabolites	Metabolites common across race, sex, and distance
Trehalose 6-phosphate	2-Isopropylmalate	Homocitrulline	UDP-glucose
D-Glyceraldehyde 3-phosphate	Trans-trans-farnesyl diphosphate	n-Carbamoyl-l-aspartate	Hydroxyphenylpyruvate
Pikatropin	Phosphothreonine	Vanillin	UMP
Guanidoacetic acid	Phenyl lactic acid	Hypoxanthine	Alpha-ketoglutarate
3-Phosphoglycerate	NADH	Deoxyinosine	Glutathione
Asparagine	Sulfolactate	Uridine	CMP
Homocarnosine	Biotin	Cholate	Malate
dTDP	dTMP	Myo-inositol	Hydroxylysine
n-Acetylornithine	n-Acetyl glutamine	Indole	Histidinol
Pyroglutamic acid	Folate	Uracil	Cystine
GMP	Carnitine	Gluconolactone	Cysteine
Succinate/Methylmalonate	Sedoheptulose 1/7-phosphate	S-Ribosyl-L-homocysteine	Adenosine 5′-phosphosulfate
Orotate	Adenine	Kynurenine	Ornithine
Phosphoenolpyruvate	Abscisate	Phenylalanine	Glucose phosphate
Glutathione sulfonamide	Ophthalmate	Nicotinate	Lysine
Glutamate	Trehalose/Sucrose	Xylose	N-Acetylglutamate
	L-Arginosuccinate	Dipicolinate	Cytosine
	Hydroxyproline	Hydroxybenzoate	AICA-riboside
	Riboflavin	Oxaloacetate	Creatine
	Aspartate	3-Phosphoserine	2-Oxoisovalerate
	Taurine	2-Aminoadipate	Lactate
	AMP/dGMP	Purine	Acetyl lysine
	Glycolate	Nicotinamide	Citrulline
	7-Methylguanosine	Histidine	Tauro deoxycholate
	Norepinephrine	Tyrosine	Inosine
	Pyridoxine	dAMP	Cystathionine
		Homoserine/threonine	Dihydroorotate
		Glutamine	
		Kynurenic acid	
		Pantothenate	
		2-dehydro-d-gluconate	
		Tryptophan	

**FIGURE 6:**
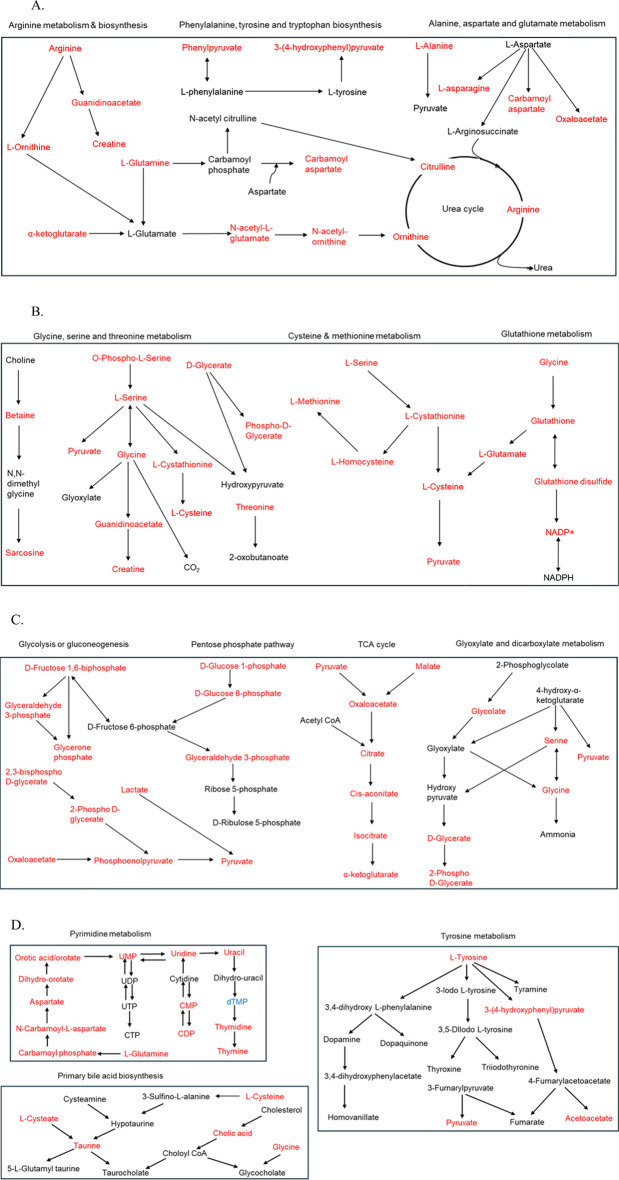
**(A)** Schematic representation of amino acid metabolism pathways enriched with metabolites that exhibit differential abundance between females and males. Metabolites highlighted in red are significantly more abundant in females, while those in blue are significantly less abundant (P < 0.1). **(B–D)** Illustrating the most impacted pathways for differentially abundant metabolites in the Less than 5 km vs. Greater than 5 km groups. Metabolites highlighted in red are significantly upregulated, while those in blue are significantly downregulated in the Less than 5 km group (P < 0.1) **(B)** Schematic representation of energy and sulfur compound metabolism pathways. **(C)** Schematic representation of energy and carbohydrate metabolism pathways. **(D)** Schematic representation of nucleotide and amino acid metabolism pathways.

Metabolic pathway enrichment analysis was performed using metabolites with a VIP score greater than 1. Pathways with an FDR <0.05 and high impact factors were selected for detailed enrichment analysis (Figures 6A–D). Metabolites associated with amino acid metabolism pathways demonstrated significant sex-based differences. Enrichment analysis revealed that metabolites such as glutathione sulfonamide, UDP glucose, geranyl diphosphate, alanine/sarcosine, guanidinoacetate, alpha-ketoglutarate, and creatine were significantly more abundant in females (P < 0.1), whereas metabolites such as lactate, glycodeoxycholate, taurodeoxycholate, 5-methyl tetrahydrofolate, and hydroxyisocaproic acid were less abundant. All significantly altered metabolites in arginine, phenylalanine, tyrosine, tryptophan, alanine, aspartate, and glutamate metabolism and biosynthesis had a higher abundance in females (Figure 6A). When comparing individuals residing less than 5 km from the TT facility to those living farther than 5 km, notable differences in energy and sulfur compound metabolism were observed (Figure 6B). Metabolites such as pyruvate, serine, alanine/sarcosine, guanidinoacetate, valine/betaine, glutathione, glutathione disulfide, glutathione sulfonamide, homoserine/threonine, cystine, cystathionine, cysteine, and tryptophan were significantly upregulated in the Less than 5 km group (highlighted in red), while phosphothreonine and abscisate were downregulated. These differences suggest potential metabolic adaptations or disruptions linked to proximity to the TT facility. Energy and carbohydrate metabolism pathways were also significantly impacted by proximity to the TT facility (Figure 6C). Metabolites upregulated in the Less than 5 km group included lactate, pyruvate, glycerate, oxaloacetate, malate, alpha-ketoglutarate, aconitate, glyceraldehyde phosphate, phosphoenolpyruvate, glycerone phosphate, and glucose phosphate (P < 0.1). Conversely, metabolites such as phenyl lactic acid and 2-isopropyl malate were downregulated. These findings highlight shifts in energy metabolism are likely driven by exposure to emissions from the TT facility. Additionally, metabolites involved in nucleotide and amino acid metabolism exhibited significant differences between the Less than 5 km and Greater than 5 km groups (Figure 6D). Red-highlighted metabolites, such as tyrosine, phenylalanine, pyruvate, cytosine, histamine, taurine, thymine, glutamine, guanine, DHPS, dihydroorotate, carnitine, cysteate, carbamoyl aspartate, hydroxyphenyl pyruvate, uridine, adenosine, guanosine, inosine, CMP, UMP, and ADP, were significantly more abundant in the Less than 5 km group (P < 0.1). In contrast, metabolites such as dTMP and 5-methyltetrahydrofolate, highlighted in blue, were less abundant. These findings indicate potential disruptions in fundamental metabolic processes in individuals residing closer to the TT facility. There were significant metabolic pathway perturbations influenced by both sex and proximity to the TT facility. Amino acid metabolism pathways showed prominent sex-based differences, while energy and carbohydrate metabolism pathways, as well as nucleotide and amino acid metabolism pathways, were most affected by proximity to the TT facility.

**FIGURE 7:**
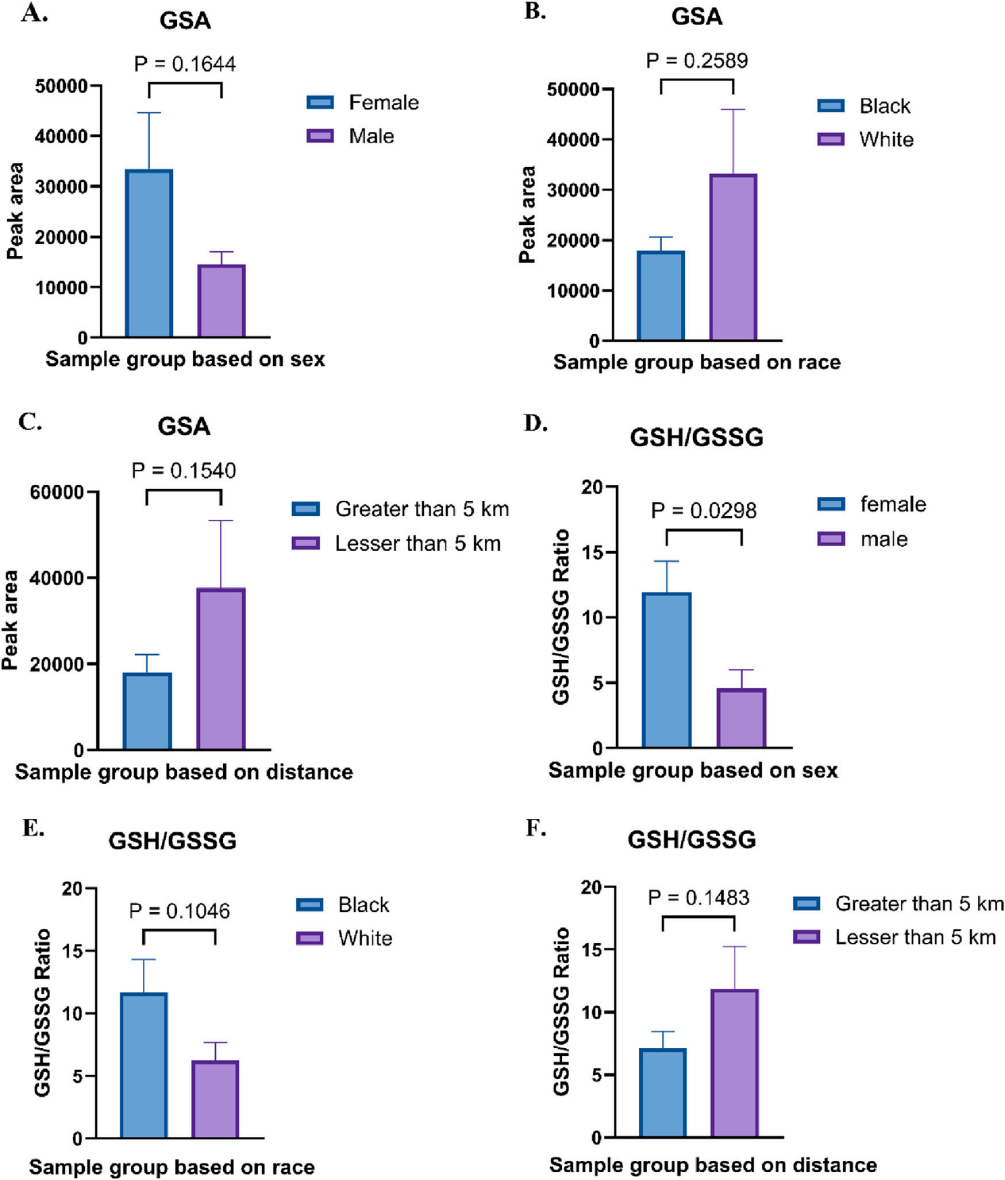
**(A)** Relative abundances of glutathione sulfonamide (GSA) in Female vs. Male sample groups. **(B)** Relative abundances of GSA in Black vs. White sample groups. **(C)** Absolute abundances of GSA in Less than 5 km vs. Greater than 5 km sample groups **(D)** Relative abundances of glutathione (GSH) and glutathione disulfide (GSSG), along with their ratio, in Female vs. Male sample groups. **(E)** GSH/GSSG abundance ratio in Black vs. White sample groups. **(F)** GSH/GSSG abundance ratio in Less than 5 km vs. Greater than 5 km sample groups.

Biomarkers for systemic inflammation and oxidative stress were evaluated across all sample groups (Figures 7A–F). The relative abundances of glutathione sulfonamide (GSA) displayed significant variations based on sex, race, and proximity to the thermal treatment facility. A study demonstrated that urinary GSA levels correlate with GSA levels in BAL and other markers of neutrophilic inflammation, suggesting that GSA could serve as a biomarker for tracking disease activity in the population [38]. Female samples exhibited higher levels of GSA compared to male samples (33,500 ± 8,337.38 vs. 14,600 ± 1,570.03) (Figure 7A). Similarly, white individuals showed significantly elevated levels of GSA compared to black individuals (18,000 ± 1812.71 vs. 33,200 ± 8,953.52) (Figure 7B). Additionally, individuals residing within 5 km of the facility exhibited higher abundances of GSA than those living farther away, indicating a potential relationship between proximity to the facility and increased metabolite levels (18,000 ± 3,161.67 vs. 37,600 ± 9,546.69) (Figure 7C). To examine antioxidant status in the groups, the glutathione/glutathione disulfide (GSH/GSSG) ratio, a critical marker of oxidative stress, was calculated across all groups. A GSH/GSSG ratio of less than 1 reflects an imbalance between reduced glutathione (GSH) and oxidized glutathione (GSSG). The relative abundances of GSH and GSSG, as well as their calculated ratio, revealed significant group-specific differences. Females demonstrated a higher GSH/GSSG ratio compared to males (18.82 ± 3.17 vs. 4.78 ± 0.79) (Figure 7D). In contrast, Black individuals exhibited elevated GSH levels and a higher GSH/GSSG ratio compared to White individuals (12.68 ± 2.57 vs. 8.96 ± 1.17) (Figure 7E). Furthermore, individuals residing closer to the thermal treatment facility (less than 5 km) displayed a slightly higher GSH/GSSG ratio compared to those living farther away (10.57 ± 1.35 vs. 11.54 ± 2.58) (Figure 7F).

## Discussion

The TT facility in Colfax, LA experienced a substantial increase in energetic waste processing volume in 2014, raising concerns about potential environmental and public health impacts from exposure to hazardous air pollutants. Residents described experiencing adverse health conditions or symptoms potentially linked to exposure to air pollutants, including thyroid disease, respiratory distress, skin lesions, hair loss and cancers [4, 39]. The metabolomics analysis of urine samples provided valuable insights into the metabolic disruptions potentially associated with environmental exposure to emissions from the hazardous waste thermal treatment facility. The identification of 217 metabolites spanning various biochemical pathways underscores the diversity of metabolic responses influenced by proximity, sex, and race. From the two-way Anova analysis, 94 metabolites out of the 217 were significantly altered by distance when compared to sex Supplementary Figure S1). Notably, the results highlight significant differences in metabolite abundance among individuals residing closest to (less than 5 km from) the facility compared to those living farther away, suggesting an environmental impact on metabolic activity.

The impact of living near thermal treatment facilities has been a subject of concern, with several studies investigating potential health effects [40–45]. These effects include cancer, respiratory diseases, cardio-cerebrovascular disease, and adverse pregnancy outcomes [40]. In this study as well, we observed that individuals residing closer to the TT facility exhibited perturbation in the pathways involved in energy and nucleotide metabolism. We observed alterations in pathways related to glycolysis, the citric acid cycle, and oxidative phosphorylation in individuals residing closer to the facility (Figure 1). These shifts may indicate altered cellular energy production and utilization, potentially impacting cellular function and overall health [46, 47]. These pathways are important in evaluating potential health risks [48]. Studies have suggested that exposure to ambient air pollution and traffic-related air pollutants is associated with dysregulated metabolism of fatty acids, amino acids, leukotrienes and glucose [49, 50]. Similarly, we observed significant alterations in carbohydrate metabolism pathways, suggesting potential disruptions in glucose utilization and energy production. Disruptions in sulfur metabolism, particularly alterations in glutathione metabolism, as observed in our study (Figure 7) suggest increased oxidative stress in individuals closer to the facility. Glutathione plays a crucial role in cellular antioxidant defense, and its dysregulation can lead to oxidative damage to cellular components [51]. We also found that nucleoside and nucleotide metabolism pathways were impacted (Figures 5, 6). Altered nucleotide metabolism could potentially impact DNA replication and repair [52]. Altered nucleotide metabolism can also contribute to cancer development and progression. Cancer cells have an increased need for nucleotides, which are used in the synthesis of DNA and RNA. This altered metabolism can help cancer cells grow quickly, resist chemotherapy, and spread to other parts of the body [53].

**FIGURE 8:**
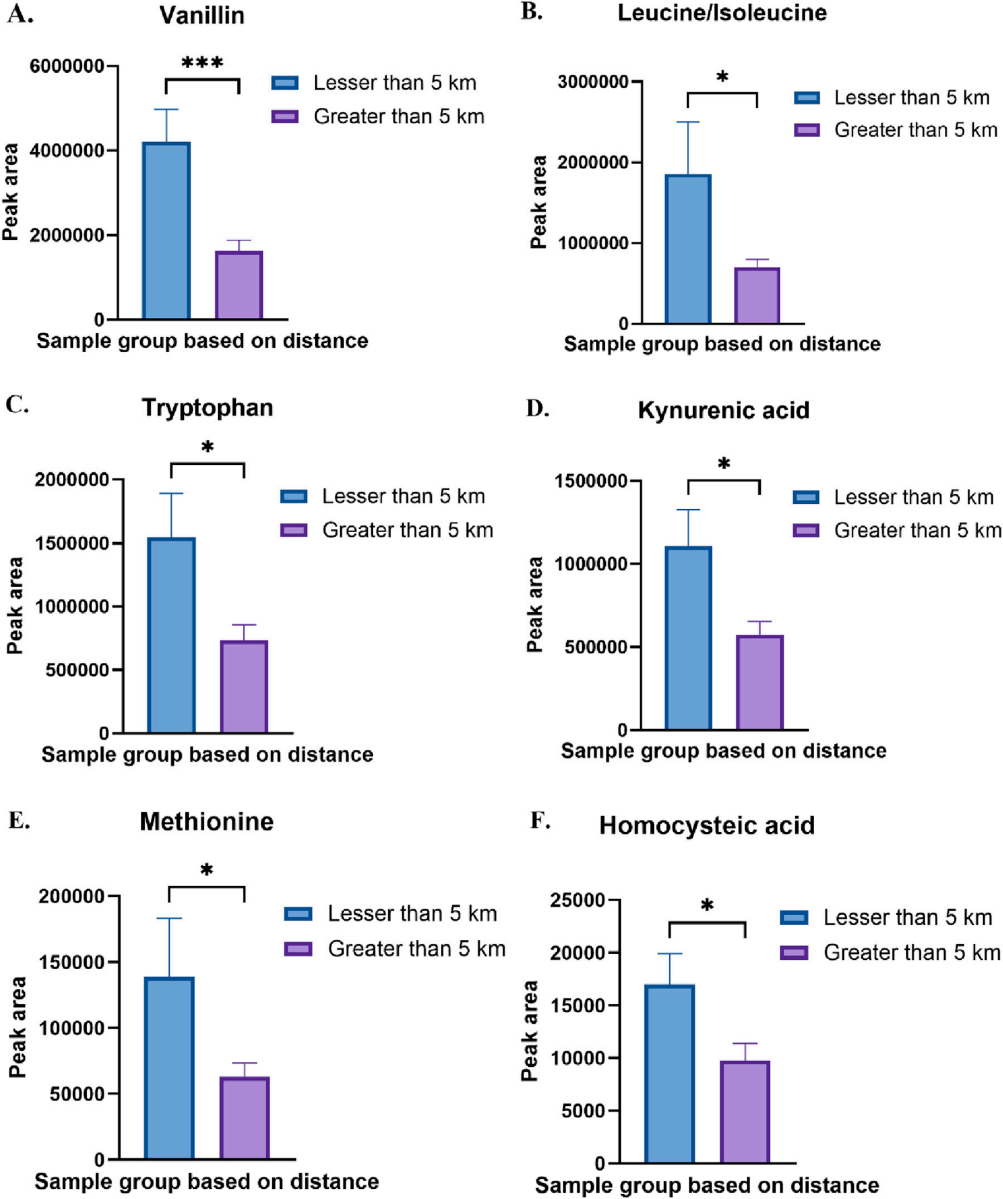
**(A)** Relative abundance of vanillin in Less than 5 km vs. Greater than 5 km sample group. **(B)** Relative abundance of leucine/isoleucine in Less than 5 km vs. Greater than 5 km sample group. **(C)** Relative abundance of tryptophan in Less than 5 km vs. Greater than 5 km sample group **(D)** Relative abundance of kynurenic acid in Less than 5 km vs. Greater than 5 km sample group **(E)** Relative abundance of methionine in Less than 5 km vs. Greater than 5 km sample group **(F)** Relative abundance of homocysteic acid in Less than 5 km vs. Greater than 5 km sample group. *p < 0.05, ***p < 0.001.

Several amino acid metabolism pathways were enriched due to the differences in distance, race and sex. Few amino acid metabolism pathways such as glycine, serine and threonine metabolism, cysteine and methionine metabolism, pyrimidine metabolism and arginine metabolism were enriched in all the three groups. Valine, leucine and isoleucine biosynthesis and phenylalanine, tyrosine and tryptophan metabolism were changed in two groups due to distance and sex. Perturbations in different amino acid metabolism pathways can have implications for cellular growth, repair, and protein synthesis. Methionine, an essential amino acid, in excess also acts as precursor for homocysteine. We observed a significant increase in methionine and homocysteic acid in individuals residing closer to the TT facility (Figure 8). Increased homocysteine or hyperhomocysteinemia is a known risk factor for cardiovascular disease, Parkinson’s disease, Alzheimer’s disease and stroke [54]. Glycine and serine are biosynthetically linked together and are essential metabolite for the survival of cancer cells [55]. Chronic dysregulation of glycine and serine metabolism can lead to cancer as these amino acids also play an important role in cellular antioxidant capacity, one-carbon metabolism and help in protein, lipid and nucleic acid synthesis. Branched-chain amino acid (BCAA) leucine, isoleucine and valine were also increased significantly in the residents due to proximity to the TT facility (Figures 1, 8). Elevated levels BCAAs observed in our study may reflect increased protein catabolism and muscle wasting, which are indicators of chronic metabolic stress. This has been consistently reported in both animal models and patients with Type 1 Diabetes Mellitus (T1DM), where plasma BCAAs are significantly elevated compared to healthy controls [56–58]. These results suggest that the elevated BCAAs in our exposed group may represent not only a consequence of environmental stress but also an early metabolic indicator of disrupted energy homeostasis. Our findings align with a previous mouse study showing that exposure to PM_2.5_-induced asthma, was associated with elevated levels of BCAAs [59]. We also found vanillin was significantly high in the residents residing closer to the TT facility (Figure 8). In a study using high-resolution metabolomics analysis of human bronchial epithelial cells exposed to vanillin, revealed disruptions in energy, amino acid, antioxidant, and sphingolipid pathways linked to lung diseases [60].

Another important amino acid pathway that was enriched due to distance and sex is tyrosine and tryptophan metabolism. We observed a significant increase in tryptophan and its metabolite kynurenic acid in the group which was close to facility (Figure 8). Tryptophan metabolism is involved in many physiological functions, including inflammation, metabolism, and immune response. Dysregulation of these metabolic pathways can contribute to the development of a number of diseases such as respiratory, nervous, digestive disorders and cancers [61]. Tryptophan and its metabolites, particularly kynurenine, can act as reactive oxygen species (ROS) scavengers by directly neutralizing ROS such as hydrogen peroxide and superoxide, converting them into less reactive or more stable compounds [62, 63]. Thus, the observed increase in tryptophan and kynurenic acid near the facility may also indicate elevated oxidative burden, given the known susceptibility of the indole ring of tryptophan to oxidation. Tyrosine is a non-essential amino acid that serves as the precursor for several crucial neurotransmitters, including epinephrine, norepinephrine, and dopamine, as well as hormone thyroxine. Dysregulation in tyrosine metabolism has been implicated in the development of several diseases, including cancers and chronic disorders [64]. Increased tyrosine in urine or dysregulated tyrosine metabolism is associated with altered thyroid function [65]. This can partially explain the increased thyroid diseases reported by the several Colfax residents [4].

We further evaluated the changes in urinary metabolites based on sex and race. Sex-specific differences revealed distinct patterns of amino acid metabolism, with females exhibiting higher levels of metabolites like glutathione sulfonamide and UDP glucose, while males displayed elevated levels of lactate and bile acids. These findings may reflect physiological differences in metabolic processing and stress response mechanisms. Similarly, race-based comparisons highlighted unique metabolic signatures, with Black individuals showing elevated levels of glutathione and a higher GSH/GSSG ratio compared to White individuals. While some of these differences may be due to inherent biological variation, they may also reflect socio-environmental disparities. Factors such as unequal exposure to environmental pollutants, differences in healthcare access, diet, and chronic stress, can influence metabolic profiles and oxidative stress responses. These observations emphasize the importance of interpreting metabolomic data within a broader social and environmental context, accounting for both biological and non-biological determinants of health.

The observed shifts in the GSH/GSSG ratio suggest potential oxidative stress and it requires detailed investigation. Under normal conditions, the GSH:GSSG ratio in mammalian cells exceeds 100:1, but this ratio decreases to 10:1 or even 1:1 under oxidative. This imbalance reflects an increase in oxidized glutathione (GSSG) relative to reduced glutathione (GSH), signifying a shift in the cellular redox state. The relative abundances glutathione sulfonamide (GSA) displayed significant variations based on proximity to the thermal treatment facility, with individuals residing within 5 km exhibiting higher abundances than those living farther away, indicating increased oxidative stress (Figure 7). Oxidative stress arises when the production of reactive oxygen species (ROS) surpasses the capacity of antioxidant defense systems, either due to insufficiency or dysfunction. This imbalance leads to significant damage to several biological macromolecules such as cellular membranes, lipids, proteins, and nucleic acids. An increase in oxidative stress is associated with onset and pathogenesis of several diseases such as thyroid disorders [66], diabetes [67], Parkinson’s disease [68], Alzheimer’s disease [69] and cancer [70]. While GSA levels and GSH/GSSG ratios are reliable biomarkers of redox status, it is important to consider potential confounding factors such as diet, medication use, renal function, and smoking, which may independently affect glutathione metabolism and oxidative balance. Future studies should incorporate detailed individual exposure histories and lifestyle data to better delineate the sources and implications of oxidative stress in environmentally exposed populations.

The study’s findings are indicative of the potential health risks associated with exposure to emissions from the TT facility. Emissions from the TT facility contain a variety of pollutants, including PM, volatile organic compounds, heavy metals and EPFRs. These pollutants can directly or indirectly interfere with cellular metabolism, leading to the observed metabolic changes. The disruption of key metabolic pathways and the association with oxidative stress biomarkers suggest that long-term exposure could lead to adverse health outcomes. Identifying unique metabolites and pathway perturbations linked to proximity highlights the need for further epidemiological studies and regulatory measures to mitigate environmental health risks.

### Limitations of the study

While the study provides compelling evidence of metabolic alterations, several limitations should be noted. First, this is an exploratory study with a relatively small sample size. However, this is a rural community, and 51 participants actually represents 3.5% of the population. Statistical analyses were performed using appropriate multivariate methods. To enhance reliability, data normalization, quality control filtering, and multiple testing corrections such as false discovery rate were also applied. For PLS-DA and VIP score analyses, model quality was assessed using Q2 values, calculated via cross-validation (CV) with thresholds above 0.5 indicating good predictive performance and model validity. Future studies involving larger cohorts and longitudinal sampling are obviously necessary to confirm these results and support their broader applicability. Power analysis for our exploratory study shows that significance of α = 0.10 could be achieved with a power of β = 0.72 for a 1.75-fold difference, which was observed for 16 compounds among those living within 5 km of the facility and 2 compounds for those living at least 5 km away. Second, due to challenges of conducting the research in a rural community (older population, low educational attainment), first-void urine samples were not collected. It is not expected that bias in the sample collection method was systematic [71]. Third, self-selection bias may have resulted in a less healthy population, especially near the TT facility, because sick participants may have been more willing to tell their stories. This error could have the potential to introduce systematic bias [72] into the results if the health of those living closer to the facility was different from those living farther away and associated with their exposure. Additionally, integrating other omics approaches, such as transcriptomics and proteomics, could provide a more comprehensive understanding of the biological impacts of environmental exposure.

## Conclusion

The metabolomic analysis of urine samples from the Colfax community members near the thermal treatment facility provides critical insights into potential environmental exposures and their impact on human health. With metabolomic profiling we found strong evidence of significant metabolic alterations in individuals residing closer to the thermal treatment facility compared to those living further away. The observed shifts in metabolite abundances and pathway perturbations, particularly in energy metabolism, oxidative stress pathways, and nucleotide and amino acid metabolism, suggest potential adverse health impacts associated with exposure to emissions from the facility. These findings highlight the importance of further investigation into the long-term health implications of these metabolic changes and the need for continued monitoring of the environmental and health impacts of the TT facility.

## Data Availability

The original contributions presented in the study are included in the article/Supplementary Material, further inquiries can be directed to the corresponding author.
