# Supplementary material for: Proximity to a hazardous waste thermal treatment facility alters human physiology: a community-driven pilot study
Source: Exp Biol Med (Maywood). 2025 Aug 15;250:10655. doi: 10.3389/ebm.2025.10655 (PMC12394085; doi:10.3389/ebm.2025.10655)
Supplement: Supplementary file 1 [file Supplementaryfile1.docx]

**Supplementary figure 1.**


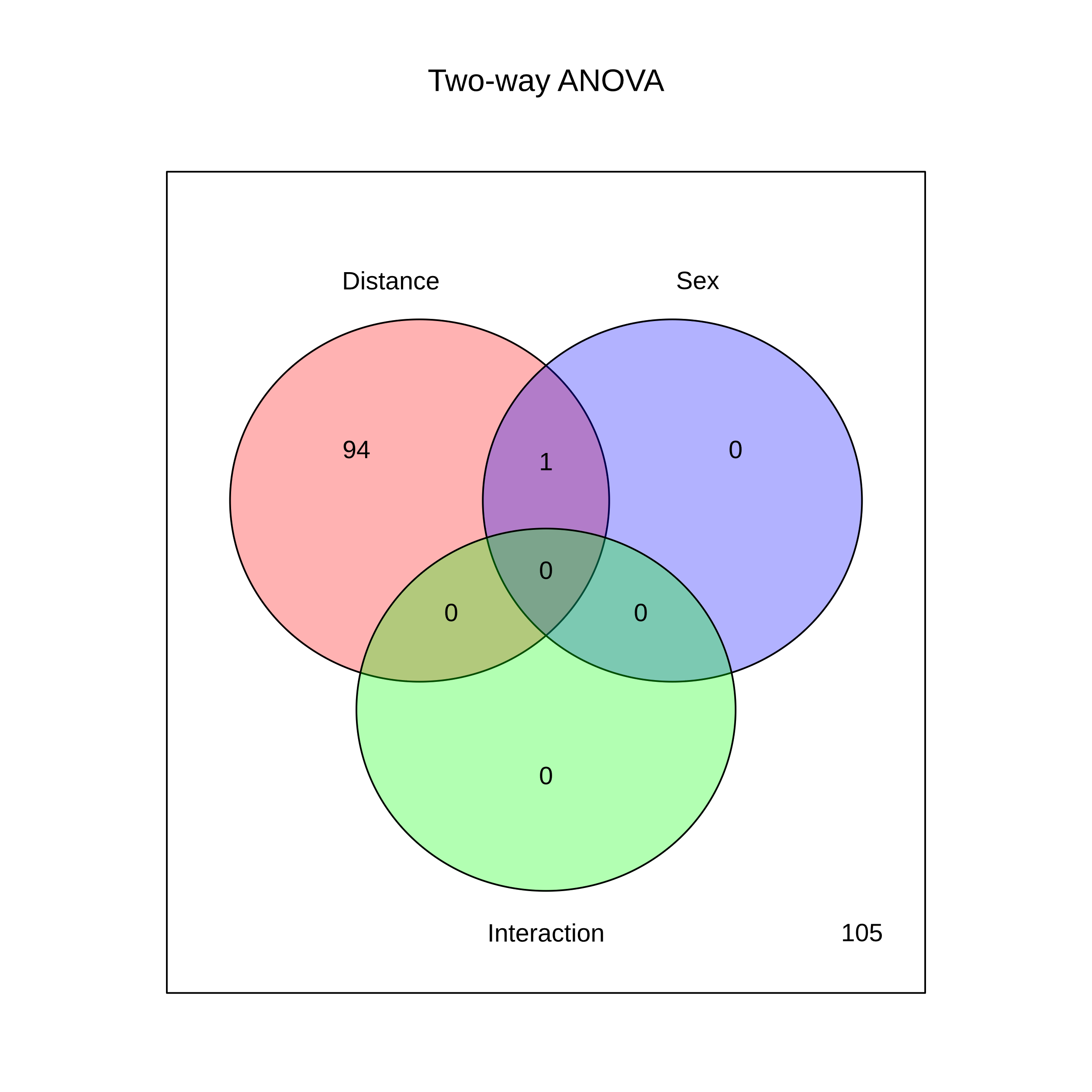


**Figure S1.** Two-way Anova plot to showing the number of metabolites that were significantly altered based on proximity only when compared with sex. (P value cutoff < 0.05).
